# Supplementary material for: Fine needle aspiration of human lymph nodes reveals cell populations and soluble interactors pivotal to immunological priming
Source: Eur J Immunol. 2024 Feb 22;54(5):2350872. doi: 10.1002/eji.202350872 (PMC13479937; doi:10.1002/eji.202350872)
Supplement: Supplementary file 1 [file EJI-54-2350872-s003.pdf]

Supporting Information Figures

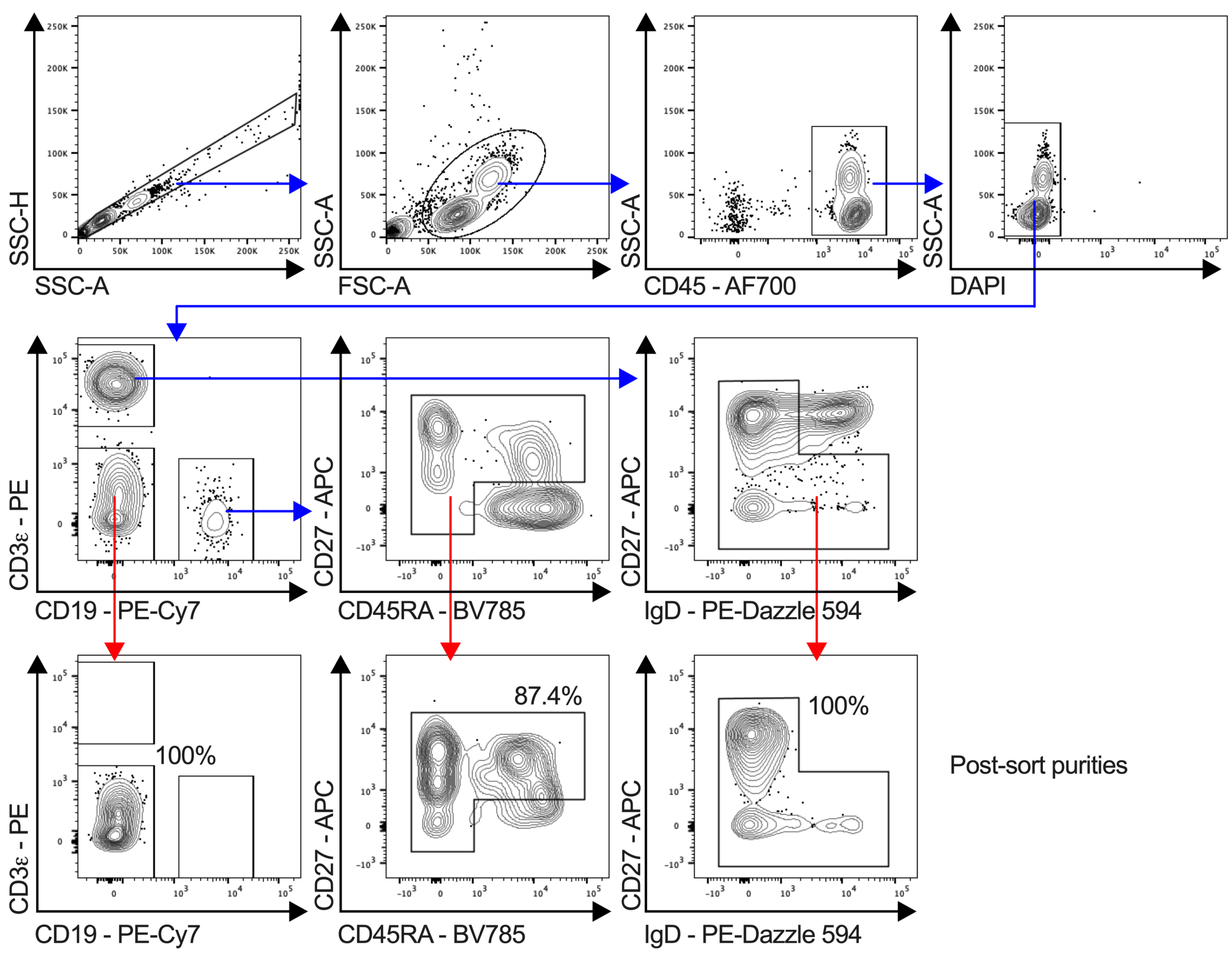

Figure S1.
Sort scheme for isolation of cells for single-cell sequencing.

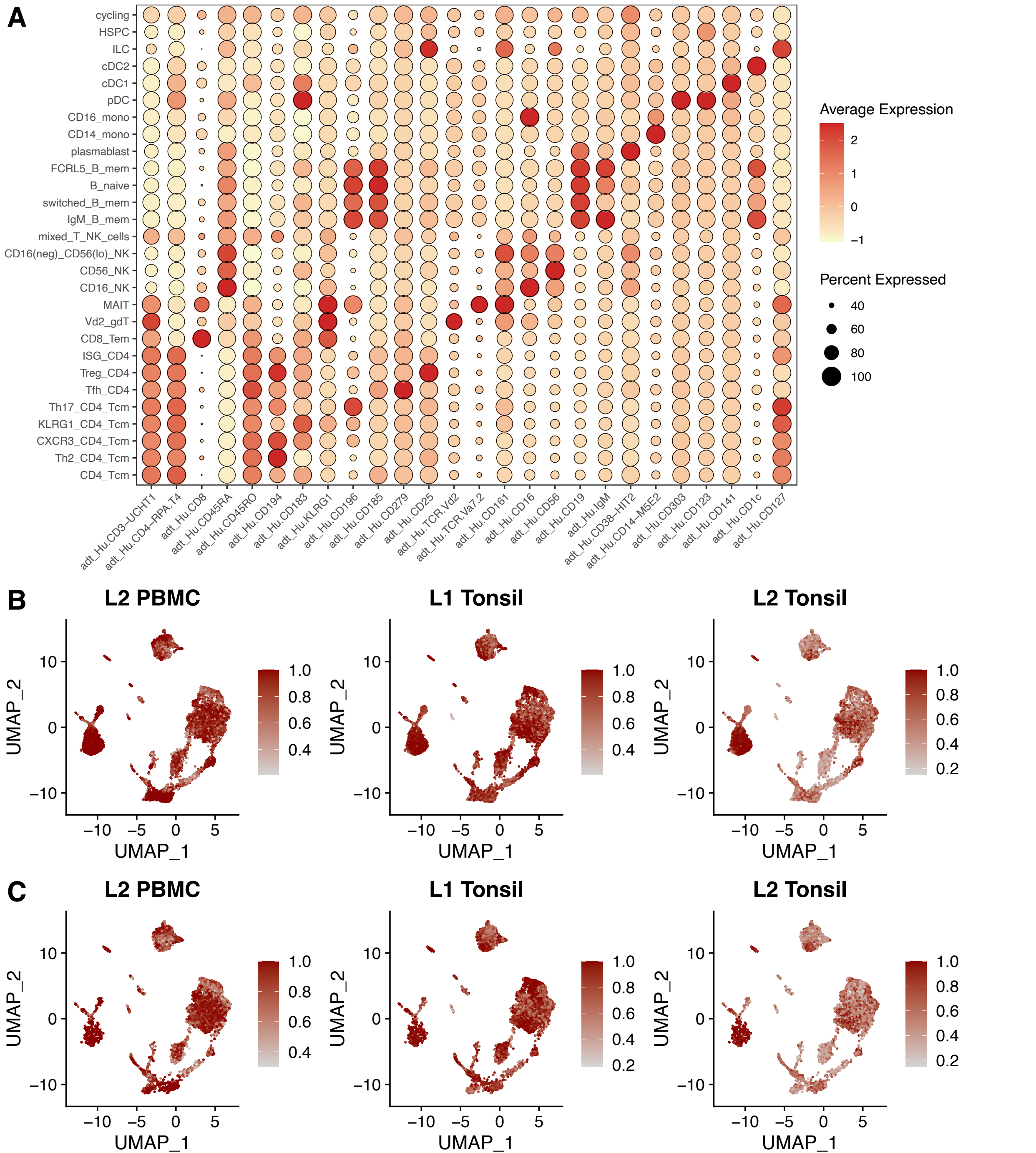

Figure S2.
(A) Dot plot showing normalized protein expression of the indicated proteins across each of the clusters. Characters after the dash (-) indicate the antibody clone for selected antibodies. **(B, C)** Annotation scores from Azimuth for blood (B) and LN (C) using the level 2 PBMC, level 1 Tonsil, and level 2 Tonsil annotations.

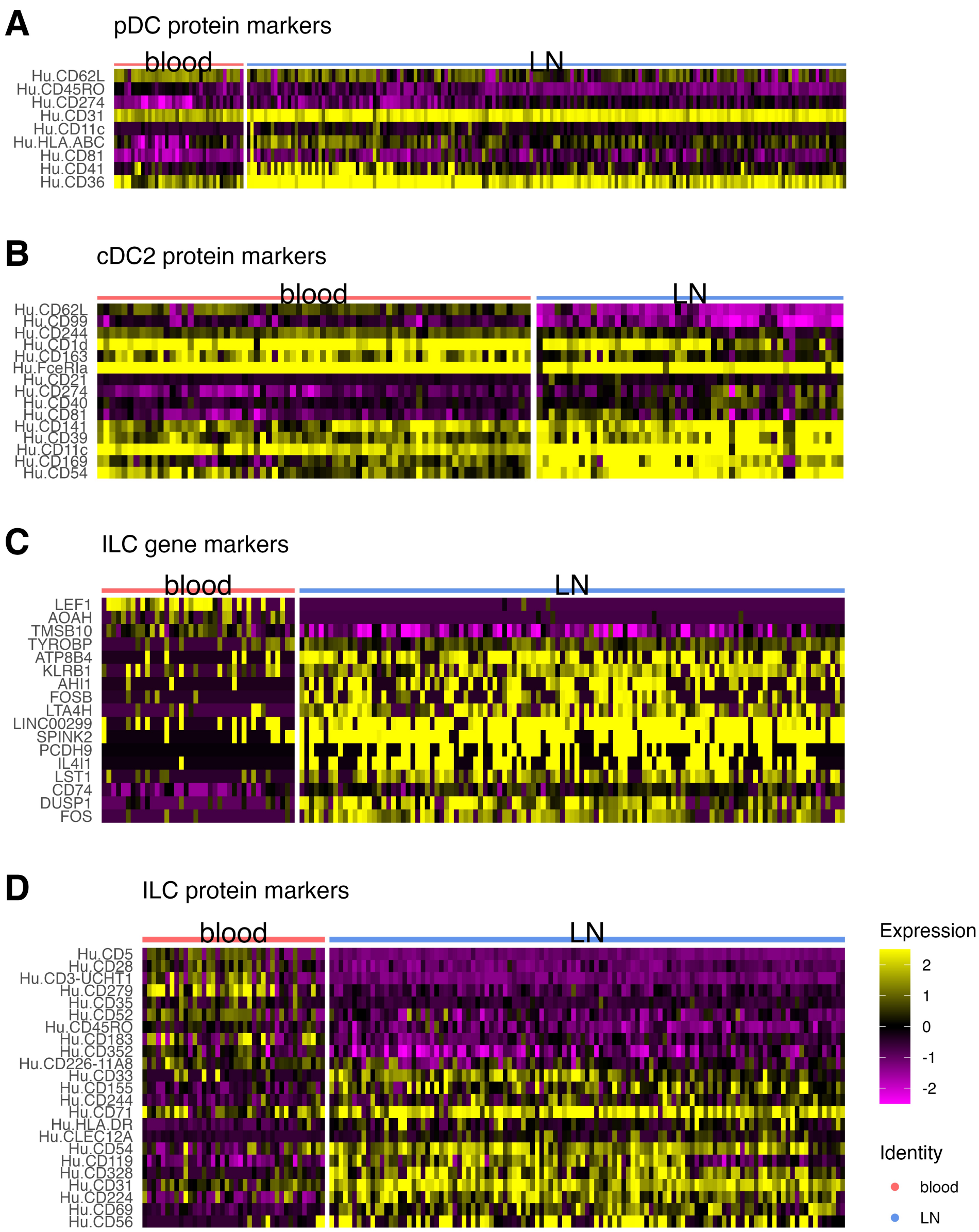

Figure S3.
(A, B) Differentially expressed proteins in pDCs (A) and cDC2s (B) between blood and LN cells. **(C, D)** Differentially expressed genes (C) and proteins (D) in the ILC cluster between blood and LN cells.

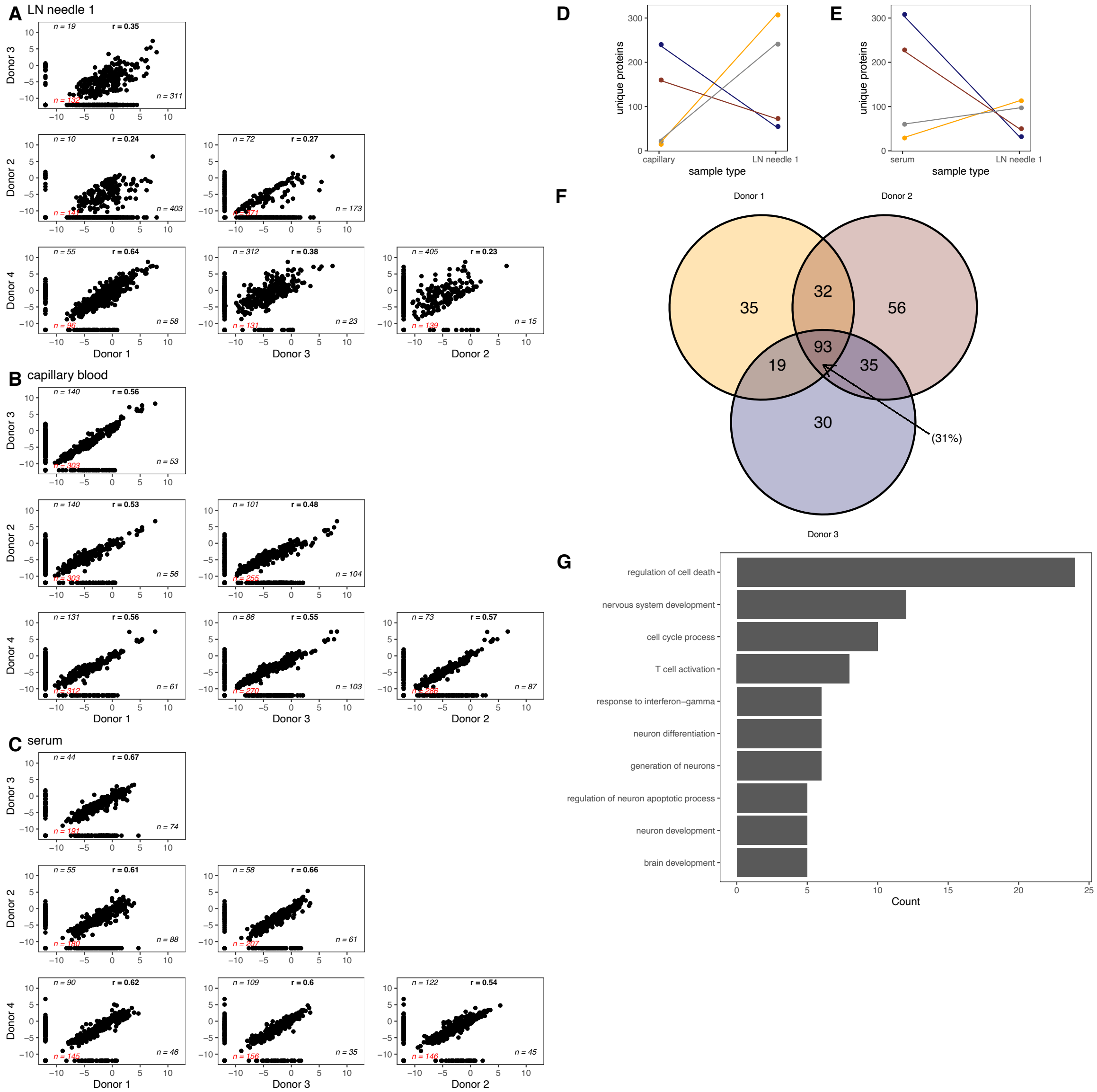

Figure S4.

(A-C) Correlation of relative protein abundance between LN FNA needle 1 (wash 1) (A), capillary blood (B), and serum (diluted 1:100) (C) for each donor. Number in black on each axis is the number of proteins not detected in the respective sample. Number in red in the bottom left is number of proteins not detected in either sample. Number in bold and black is Pearson correlation coefficient. **(D, E)** Number of proteins specifically detected in each matched sample of (D) capillary blood versus LN needle 1 (wash 1) or (E) serum (diluted 1:100) versus LN needle 1 (wash 1). Each color represents a donor. **(F)** Venn diagram of overlap of proteins specifically detected in each of the LN needle 2 (wash 1) samples compared to matched serum (diluted 1:100). Number in parentheses indicates percent of proteins found in all three samples. **(G)** GO pathway analysis (ontogeny = biological process) of the 92 proteins specifically found in all three LN needle 2 (wash 1) samples compared to matched serum (diluted 1:100). Selected pathways are shown. Count is number of proteins from the dataset contributing to each pathway.
